# Impact of specialty and level of training on CT measurement of femoral version: an interobserver agreement analysis

**DOI:** 10.1007/s10195-013-0263-x

**Published:** 2013-08-29

**Authors:** Richard S. Yoon, John D. Koerner, Neeraj M. Patel, Michael S. Sirkin, Mark C. Reilly, Frank A. Liporace

**Affiliations:** 1 Division of Orthopaedic Trauma, Department of Orthopaedic Surgery, NYU Hospital for Joint Diseases, 301 E 17th Street, Suite 1402, New York, NY 10003 USA; 2 Division of Orthopaedic Trauma, Department of Orthopaedic Surgery, UMDNJ, New Jersey Medical School, 90 Bergen Street, Suite 1200, Newark, NJ 07101 USA

**Keywords:** Interobserver, Femoral version, Radiology, Level of training

## Abstract

**Background:**

To determine the interobserver agreement on femoral version measurements between an orthopedic attending, orthopedic senior and junior residents, and an attending radiologist.

**Materials and methods:**

Postoperative computed tomography (CT) scanograms of 267 patients who underwent femoral intramedullary (IM) nailing with corresponding radiology attending reads for femoral version were collected and de-identified. Femoral version measurements performed by a trauma fellowship-trained attending orthopedic surgeon (ORTHO), a senior orthopedic resident (PGY4), a junior orthopedic resident (PGY1), and a musculoskeletal fellowship-trained attending radiologist (RADS) were compared via Pearson’s interclass correlation coefficient to assess interobserver level of agreement.

**Results:**

Version measurements provided by the two attending physicians exhibited the highest level of agreement (*r* = 0.661, *p* < 0.01). The orthopedic attending and the senior resident had the next highest level of agreement (*r* = 0.543, *p* < 0.01). The first-year orthopedic resident had the weakest agreement across the board: with the orthopedic attending, the radiology attending, and the senior resident.

**Conclusion:**

Regardless of specialty, experience and higher levels of training produce stronger agreement when measuring femoral version. Residents in training, especially those who are junior, produce weak agreement when compared to their senior colleagues.

**Level of evidence:**

Level III, diagnostic study.

## Introduction

Anterograde and retrograde intramedullary (IM) nailing is a reliable, well-accepted treatment modality for a wide variety of femur fractures [1–4]. However, malrotation, occurring in 17 % to over 30 % of cases, is considered the most difficult parameter to control [2, 3, 5–12]. Many techniques have been described to assess intraoperative and postoperative rotation, including clinical evaluation, ultrasound, fluoroscopy, and computed tomography (CT), each with its proponents and critics [3, 5, 6, 8, 13–23].

While the reliability and reproducibility of CT scan version measurements have been questioned, this imaging modality is still commonly used to assess femoral length and version after IM nailing, especially in higher-energy injuries with significant comminution [3, 6, 11, 17, 20, 24]. Quantitative measurements of femoral version may also vary depending on characteristics of the observer, including specialty (radiology versus orthopedic surgery) and level of training. To our knowledge, there are no reports comparing the interobserver agreement on CT scanogram measurements of femoral version between specialties and levels of training. Thus, the focus of the study described in the present paper was to measure and assess the interobserver agreement between measurements provided by orthopedic surgeons, at various levels of training, and an attending radiologist.

## Materials and methods

All human and animal studies were approved by the appropriate ethics committee and were therefore performed in accordance with the ethical standards laid down in the 1964 Declaration of Helsinki and its later amendments; informed consent was waived and not required by our IRB. All data were collected retrospectively in conjunction with the Orthopaedic Trauma Femoral and Tibial Intramedullary Nail Registry. Study cohort formulation was determined according to specific inclusion and exclusion criteria with a subsequent registry search. Inclusion criteria included complete study records in regards to baseline and demographic data (age, gender, BMI, mechanism of injury, fracture side, open or closed, nail type—antegrade or retrograde) and availability of a CT scanogram with a corresponding version measurement performed and dictated by a musculoskeletal fellowship-trained attending radiologist. Those patients without completed chart data and/or available CT scanograms, or those with CT scanograms but without corresponding radiologist version measurements, were excluded from this study.

**Fig. 1:**
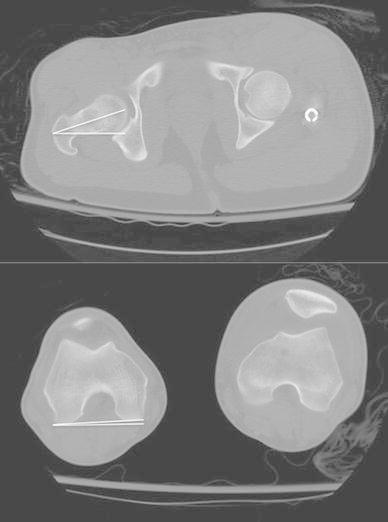
The first measurement is a result of a line drawn through the axis of the femoral neck and referenced to the horizontal. The next measurement is a second line drawn tangential to the posterior aspect of the femoral condyles, and again referenced to the horizontal. Subtracting the distal angle from the proximal angle gives the final femoral version calculation

Following study cohort formulation, a third-party research assistant (RSY) collected all corresponding postoperative CT scanograms, which were subsequently de-identified and electronically saved in a password-protected folder on a single, dedicated picture archiving and communication system (PACS) viewing station. Participants remained blinded and included an orthopedic trauma fellowship trained attending physician (ORTHO), a senior orthopedic resident (PGY4), and a first-year orthopedic intern (PGY1); participants were not allowed to view any associated dictated reports attached to the PACS image set. The same blinded, third-party researcher (RSY) obtained final version determinations collected from dictated reports which were performed by a musculoskeletal fellowship-trained attending radiologist (RAD). All measurements were completed as described by Jeanmart et al. and modified by Dugdale et al., utilizing the femoral necks and femoral condyles to calculate version (Fig. 1) [20, 23]. Participants were required to complete all measurements within 2 weeks of the start, on the same PACS viewing machine. All measurements were compiled and stored via Microsoft (Redmond, WA, USA) Excel.

Statistical analysis was performed via SPSS 18.0 (IBM Corp., Armonk, NY, USA). Interobserver agreement was compared via Pearson’s correlation coefficient (*r*), which was determined as the most appropriate statistical test for continuous data measured by different entities to calculate linear correlation. Pearson’s correlation coefficient (*r*) is correctly interpreted by assessing the calculated coefficient in the range between −1 and 1. Agreement is strongest when the coefficient is equal to 1 or −1 and is weakest when equal to 0. Significant agreement was considered to correspond to a *p* value <0.05.

## Results

From December 2000 to August 2009, 417 patients sustained femur fractures and were treated definitively via intramedullary nail. Of those, 267 patients met the inclusion criteria and formulated the study cohort for subsequent analysis.

Mean age was 31.2 ± 13.4 years with an approximately 5:1 male to female ratio. Mean BMI was 27.4 ± 5.4. The majority of our cohort were of African-American ethnicity (57.3 %), followed by Caucasian (21.0 %) and Hispanic (19.5 %). Most of the patients sustained their femur fractures secondary to motor vehicle accidents (45.7 %) or as a pedestrian struck by a vehicle (21.0 %). Other mechanisms of injury included gunshot wounds (12.0 %), a high-energy fall (10.5 %), or motorcycle accident (7.5). Less common mechanisms included crush and assault injuries (Table 1).

**Table 1:** Baseline and demographic study cohort characteristics (*n* = 267)

Mean age, years (SD)	31.2 (13.4)
Gender	
Male (%)	220 (82.5)
Female (%)	47 (17.5)
Mean BMI (SD)	27.4 (5.4)
Ethnicity	
African-American/Black (%)	153 (57.3)
White (%)	56 (21.0 %)
Hispanic (%)	52 (19.5 %)
Asian/other (%)	6 (2.2)
Mechanism of injury	
Motor vehicle accident (%)	122 (45.7)
Pedestrian struck (%)	56 (21.0)
Gunshot wound (%)	32 (12.0)
Fall (%)	28 (10.5)
Motorcycle accident (%)	20 (7.5)
Crush injury (%)	7 (2.6)
Assault (%)	2 (0.7)
Fracture side	
Right (%)	147 (55.1)
Left (%)	117 (43.8)
Bilateral (%)	3 (1.1)
Closed injury (%)	234 (87.6)
Open injury (%)	33 (12.4)
Anterograde (%)	174 (65.1)
Piriformis start (%)	110 (63.2)
Trochanteric start (%)	64 (36.8)
Retrograde (%)	93 (34.8)

Fractures occurred relatively proportionally when comparing left and right, with few bilateral injuries. The vast majority of the patients sustained closed injuries (87.6 %). Surgically, most of the patients were definitively treated via anterograde IM nails (65.1 %), usually piriformis fossa entry nails (63.2 %, Table 1).

Statistical analysis yielded strong agreement regarding the version calculations determined by attending physicians in different specialties (ORTHO vs. RAD: 0.661, *p* < 0.01), while less agreement was found with the attending radiologist’s measurements as the level of training decreased from PGY4 (PGY4 vs. RAD: 0.477, *p* < 0.01) to PGY1 (PGY1 vs. RAD: 0.139, *p* < 0.05, Table 2).

**Table 2:** Interobserver agreement between femoral version determination, as evaluated via Pearson’s correlation coefficient (*r*), and the mean difference (°) between observers along with the standard deviation of this difference, which indicates interobserver variance

Measurements compared	Pearson’s correlation coefficient (*r*)	Mean difference (SD)
ORTHO vs. RAD	0.661**	4.1 (4.4)
PGY4 vs. RAD	0.477**	4.9 (6.9)
PGY1 vs. RAD	0.139*	7.5 (6.6)
ORTHO vs. PGY4	0.543**	4.9 (6.4)
ORTHO vs. PGY1	0.061	7.7 (7.3)
PGY4 vs. PGY1	0.110	8.2 (8.1)
Ortho total^a^ vs. RAD	0.599**	4.3 (4.0)

^a^Ortho total = mean measurement of ORTHO, PGY4, PGY1

** *p* < 0.01; * *p* < 0.05

Regarding agreement amongst those in orthopedic surgery, strong correlation was found between measurements taken by the attending and senior resident (ORTHO vs. PGY4: 0.543, *p* < 0.01). Weak, although not statistically significant, agreement was found between the version determinations made by the attending and senior resident when compared to the PGY1, respectively (ORTHO vs. PGY1: 0.061, PGY4 vs. PGY1: 0.110, *p* > 0.05, Table 2). When the calculations of those at all orthopedic training levels were averaged, these mean version measurements exhibited a relatively strong, significant agreement with the measurements of the radiologist (ORTHO TOTAL vs. RAD: 0.599, *p* < 0.01, Table 2).

Investigating the interobserver variance, the mean difference and the standard deviation of it also correlated with the level of training. The mean difference remained lower than the threshold of clinical significance amongst the more senior observers, while more inexperienced observers exhibited more erratic outcomes (Table 2).

## Discussion

Malrotation is a dreaded and, unfortunately, common adverse event following IM nailing of the femur [1, 5, 9]. Several methods have been developed in order to avoid this outcome [3, 5–7, 14, 17, 22, 25–27]. For simple fracture patterns, intraoperative fluoroscopy can be utilized to obtain optimal cortical alignment or compare the injured side to the contralateral extremity [6, 8, 27]. However, for higher-energy fractures often associated with significant degrees of comminution, postoperative CT is a useful tool to confirm proper rotational alignment [6, 20, 28].

To our knowledge, our study is one of the first in the literature to assess interobserver agreement in measured femoral version between orthopedic surgeons at various levels of training and an attending radiologist. Not surprisingly, measurements by those at higher levels of training exhibited the highest levels of interobserver agreement. Regardless of specialty, experience seemed to play an important role in providing agreeing data, as the PGY1 reported the lowest agreement with any of his senior colleagues. Perhaps even more critical was the trend noted in comparative mean differences. As more experienced observers were compared, they reached the threshold of clinical significance (3–4 degrees). This indicates that even a Pearson’s value that correlates with poor agreement would denote an acceptable value.

In general, CT is an accurate and reliable imaging modality, especially for bony visualization and rotational measurements. In the scoliosis literature, it has been utilized to assess axial vertebral rotation with high accuracy and low variability, with studies showing variability of only 3–5° amongst observers [29, 30]. Similarly, CT has been a trusted modality in the measurement of femoral version amongst orthopedic traumatologists [6, 20, 28]. Dugdale et al. [20] first described its value in identifying and planning for corrective osteotomy following femoral malrotation. Since then, CT has been the standard for comparisons aimed at determining the usefulness of fluoroscopy as well ultrasound in the assessment of femoral version [6, 20, 28]. Furthermore, as we move forward into the twenty-first century, new innovations and melds of technology are becoming more apparent in the orthopedic realm. In a cadaveric study, Hawi et al. [31] noted a novel method of measuring femoral neck anteversion via the use of a smartphone device. Version measurements also were accurate and were confirmed through comparison with CT measurements [31].

However, the literature is scarce regarding the accuracy, reproducibility, and interobserver agreement of CT in the measurement of femoral version [23, 24]. Jaarsma et al. tested the reproducibility of measurements taken by an orthopedic attending surgeon, an orthopedic resident, and an attending radiologist, and found relatively low intraobserver variance, ranging from 2.5° to 4.5°. However, when asked to perform multiple measurements on the same image set, the ability to repeat consistent measurements was poor [24]. It is important to note that while this study tested the reproducibility of Jeanmart’s method amongst three different observers, the authors did not analyze or report interobserver agreement, as was performed in our study [23, 24].

Our study is not without its limitations. While version measurements calculated by the orthopedic surgeons were done in a systematic, prospective fashion, the radiologist’s version determinations were retrieved retrospectively from available dictated reports. Radiologists were not asked to participate in a prospective fashion due to the limitations of our institution’s PACS software; while it allowed for de-identification, it did not allow for the detachment of dictated reports. Thus, with a radiology read and calculation already available, the ensuing bias could not be removed without significant individual supervision.

Furthermore, while agreement by statistical definition was considered to be strong amongst the measurements determined, there was clearly room for higher interobserver correlation. Higher levels of agreement could have been achieved by PACS software that allowed for superimposition of the femoral head and neck on the shaft, or via a more systematic methodology. In their study, Jaarsma et al. hypothesized that the lack of reproducibility, even amongst individual raters, could have been a result of a lack of consistent identification of the optimal axial femoral neck cut. Standardizing that view and measurement alone would represent a useful future study and further tighten inter- and intraobserver reliability, reproducibility, and agreement amongst tested raters [24].

Our study suggests that increasing levels of experience yields increasing agreement among femoral version measurements following IM nailing. Regardless of specialty, the attending physicians showed significantly strong agreement, while the more junior members of the team exhibited less agreement. However, while this agreement was strong, it could have been better. This calls into question the individual reproducibility of determinations of femoral version via CT, as indicated by Jaarsma et al. [24]. Future studies are required in order to develop the most accurate, reliable, and reproducible method of determining femoral version via CT scan.
